# Chikungunya, a new threat propagated by the cosmopolite *Aedes albopictus*

**Published:** 2011-01-10

**Authors:** M Vazeille, E Martin, L Mousson, AB Failloux

**Affiliations:** 1 Molecular Genetics of Bunyavirus, Institut Pasteur, F-75724 Paris Cedex 15, France

## 

The recent outbreaks of chikungunya (CHIK) were due to an East-Central-South African genotype harbouring a substitution from an Alanine (E1-226A) to a Valine (E1-226V) at the position 226 in the E1 gene. The new variant E1-226V is very efficiently transmitted by an unusual mosquito vector, *Aedes albopictus*. We found that (i) *Ae. albopictus* females ensure a high replication rate of E1-226V up to 10^9^ viral particles per female, (ii) the midgut plays a key role in limiting viral dissemination of E1-226A in the mosquito, (iii) the virus is detectable in the saliva as soon as two days after the infectious blood-meal, and (iv) *Ae. albopictus* is able to deliver up to 3000 viral particles with its saliva. All these characteristics led to exacerbate CHIK transmission by *Ae. albopictus*. However, this species is affected by the viral infection. Indeed, CHIK infection reduces sharply the survival of *Ae. albopictus*, females laying their eggs just before dying. Females did not die from an excess of viral replication but more likely in attempting to mount an immune antiviral response. Moreover, by removing the intracellular bacteria *Wolbachia* from *Ae. albopictus* through successive antibiotic treatments, we aimed to determine if *Wolbachia* interferes with CHIK replication in the mosquito. We found that *Ae. albopictus* cleaned of *Wolbachia* was not affected by CHIK infection. Thus, *Wolbachia* may regulate viral replication in *Ae. albopictus* with consequences on its survival. So, the response of a vector to a particular pathogen is also closely linked to the presence of other microorganisms. Finally, we showed that *Ae. albopictus* was able to be orally co-infected with CHIK and dengue (DEN) viruses and to deliver concomitantly infectious particles of both viruses in saliva. This finding is of particular concern as *Ae. albopictus* is still expanding its geographical range and as both CHIK and DEN viruses can co-circulate in the same geographical regions. Indeed, reports of co-infections in patients with both viruses are increasing.

